# Supplementary material for: Collaborative intelligence in AI: Evaluating the performance of a council of AIs on the USMLE
Source: PLOS Digit Health. 2025 Oct 9;4(10):e0000787. doi: 10.1371/journal.pdig.0000787 (PMC12510544; doi:10.1371/journal.pdig.0000787)
Supplement: S2 File — (ZIP) [file pdig.0000787.s002.zip › S2 Data Raw data files containing unprocessed question inputs and LLM outputs including transcripts of deliberations/STEP3_Question-10-1_Type-MC-NJ.docx]

**Facilitator** **[Round 1]:** A 47-year-old man is admitted to the intensive care unit because of lower gastrointestinal bleeding. He has a history of alcohol-induced cirrhosis. Physical examination shows marked ascites and splenomegaly; the spleen tip is palpated 4 cm below the left costal margin. Results of laboratory studies are shown: Serum Blood Creatinine 1.5 mg/dL Hematocrit 29% Hemoglobin 9.7 g/dL Platelet count 105,000/mm³ PT 25 seconds INR 2.5 Which of the following mechanisms most increases this patient’s risk for bleeding? (A) Decreased production of factor VII (B) Defective platelet aggregation (C) Deficiency of thrombopoietin (D) Inhibition of vitamin K epoxide reductase (E) Splenic sequestration

**AI_B:** RESTATING THE QUESTION: The key question here is what might be the primary underlying mechanism that is enhancing this 47-year-old male patient's risk for bleeding, particularly given his alcohol-induced cirrhosis, lower gastrointestinal bleeding, marked ascites and splenomegaly, and the lab results as provided. PLANNING: We have a set of data points to consider, but they may not all be entirely relevant. The main aspects we'll focus on are his alcohol-induced cirrhosis and the lab results, taking into account particularly the prolonged Prothrombin Time (PT) and increased International Normalized Ratio (INR), both indicative of impaired liver function. CLINICAL REASONING: Many elements can influence bleeding risk. Of the options provided, lower production of factor VII, defective platelet aggregation, deficiency of thrombopoietin, inhibition of vitamin K epoxide reductase, and splenic sequestration could all contribute. However, given the presence of alcohol-induced cirrhosis, one of the main concerns would be the liver's substantially reduced capacity to synthesize coagulation proteins. One of these is factor VII, which is a vitamin K-dependent clotting factor. The patient's prolonged PT and increased INR further suggest a deficiency in the vitamin K-dependent coagulation pathway. So option (A), "Decreased production of factor VII," seems like a potential key contributor here. But we should also consider option (D), "Inhibition of vitamin K epoxide reductase," as alcohol can inhibit vitamin K absorption in the gut, leading to a deficiency and reduced ability to synthesize coagulation factors. CONCLUDING: While factor VII deficiency could be a contributor to the bleeding, the root cause in this case is more likely to be a broader inhibition in the coagulation pathway via vitamin K deficiency, given the presence of alcohol-induced cirrhosis and the tested prolongation of PT and INR. RESPONSE: Therefore, the mechanism that most increases this patient’s risk for bleeding is (D) "Inhibition of vitamin K epoxide reductase," which could be attributed to chronic alcoholism leading to diminished absorption of vitamin K in the gut and ultimately impacting the synthesis of vitamin K-dependent clotting factors, including factor VII, in the liver.

**AI_C:** (1) RESTATING THE QUESTION: So, the patient in question is a 47-year-old male with a history of alcohol-induced cirrhosis who's been admitted to the ICU due to lower gastrointestinal bleeding. On examination, he's got significant fluid accumulation within his abdomen (ascites), and an enlarged spleen (splenomegaly). Additionally, his lab results show elevated creatinine, low hematocrit and hemoglobin values, decreased platelet count, and prolonged PT and elevated INR - all indicative of liver dysfunction and coagulation disorders. The question is asking which of the given mechanisms could be the primary cause increasing this patient's tendency for bleeding. (2) PLANNING: It's crucial to identify that both liver dysfunction (due to alcohol-induced cirrhosis) and splenomegaly can contribute to various coagulation disorders and affect platelet count. The listed options correspond to these issues in different ways. However, we could question if other factors such as the reduced platelet count being caused by splenic sequestration or decreased production of clotting factors like factor VII due to liver cirrhosis could be of primary significance. (3) CLINICAL REASONING: In this patient's case, the liver damage due to alcohol abuse has likely led to a decreased production of clotting factors, such as factor VII. This is supported by the patient's prolonged PT and increased INR, which suggest a deficiency in the production of these factors. However, one must also consider that the problem can be compounded by the enlarged spleen. The spleen's role in this context is to filter and destroy old cells, including old platelets. When the spleen becomes enlarged (splenomegaly) as seen in this patient, it could potentially sequester too many platelets, lowering the overall number available to help stop bleeding. (4) CONCLUDING: Looking at this patient's history, tests, and symptoms, his increased bleeding risk could be attributed to significantly lowered platelet count which signifies poor clotting ability, which is a consequence not simply attributable to the liver's decreased capability of producing clotting factors, but also the splenic sequestration caused by the enlarged spleen. (5) RESPONSE: Therefore, the mechanism most increasing this patient's risk for bleeding is 'Splenic sequestration' (Option E).

**AI_D:** RESTATING THE QUESTION: The information given describes a 47-year-old man with a history of alcohol-induced cirrhosis, who presents with signs of lower gastrointestinal bleeding. He has a few significant signs such as ascites and splenomegaly, reflected on physical examination, and laboratory findings that include elevated creatinine, reduced hematocrit and hemoglobin, relatively lower platelet count, and prolonged prothrombin time (PT) and international normalized ratio (INR). The question asks me to determine the potential pathological mechanism that is most likely increasing this patient's risk for bleeding out of the given options. PLANNING: The options given are as follows: A) Decreased production of factor VII; B) Defective platelet aggregation; C) Deficiency of thrombopoietin; D) Inhibition of vitamin K epoxide reductase; E) Splenic sequestration. Naturally, we would need to consider that in a bleeding patient the imbalance between coagulation factors and platelets (natural anticoagulants and clot breakers) are key, and all the choices are related to these possibilities. CLINICAL REASONING: This patient suffers from chronic liver disease (CLD), induced by prolonged alcohol consumption. The liver is primarily responsible for the synthesis of most plasma proteins including clotting factors and thrombopoietin. Therefore it's plausible that liver damage could lead to a decrease in the production of these proteins. However, the patient also shows splenomegaly - which can result in the sequestration of platelets, hence reducing the availability of these platelets for normal hemostasis. Now weighing these options, we notice that the patient's PT and INR are prolonged which suggests a defect in the clotting cascade that could be related to a decrease in the production of Vitamin K dependent clotting factors (II, VII, IX, and X). But, his platelet count, though on the lower side, are not severely reduced. CONCLUDING: Considering all the given factors, the patient's prolonged PT and INR are best explained by the decreased production of clotting factors as a result of liver damage. Although platelet sequestration in the spleen and decreased thrombopoietin production could contribute to the bleeding, the most prominent and directly measurable manifestation related to bleeding risk in this case seems to be associated with abnormal coagulation tests. The clotting factor that is responsible for increased PT and INR and that is also produced in the liver where damage has been caused due to alcohol-induced cirrhosis is Factor VII. RESPONSE: Thus, the most potential mechanism that increases this patient’s risk for bleeding is (A) Decreased production of factor VII.

**AI_A:** RESTATING THE QUESTION: According to the provided clinical scenario, a 47-year-old man with a history of alcohol-induced cirrhosis has been admitted to the ICU due to lower gastrointestinal bleeding. His physical assessment reveals severe ascites and an enlarged spleen. He also has a lower hematocrit, hemoglobin, and platelet count and an elongated prothrombin time (PT) with increased INR. We are required to determine what mechanism is primarily contributing to his increased risk of bleeding. The options given range from coagulation factor deficiencies to platelet issues and involve complex processes like thrombopoiesis and vitamin K-dependent clotting factor synthesis. These are components of the hemostatic system, ensuring that blood remains fluid in the vessels but can also clot to prevent excessive bleeding. PLANNING: The patient's risk of bleeding could be influenced by numerous factors, including decreased production of factor VII that would impair the extrinsic clotting pathway; defective platelet aggregation which is essential to form a clot; deficiency of thrombopoietin, a hormone that regulates platelet production; inhibition of vitamin K epoxide reductase, an enzyme critical for the function of vitamin K dependent clotting factors (II, VII, IX, X, protein C, and S); or splenic sequestration, a process whereby the enlarged spleen can trap and destroy platelets, reducing their availability in the circulation. However, considering the patient's alcohol-induced cirrhosis and physical examination findings of marked ascites and splenomegaly, the primary clinical assumption would be compromised liver function and its associated coagulopathy, combined with the potential for splenic sequestration due to splenomegaly. CLINICAL REASONING: In patients with alcoholic cirrhosis like our patient, hepatic synthetic function is often compromised. Given that the liver produces thrombopoietin and clotting factors, including VII, any liver damage could affect the production of these entities. However, the patient's platelet count, while possibly lower due to splenic sequestration, is not abnormally low. The inability to produce clotting factors would also reflect in the patient's PT and INR values, both of which are prolonged. This suggests a problem with the liver's ability to produce clotting factors and is backed up by the alcohol-induced cirrhosis' story. The patient's cirrhosis could also lead to secondary hypersplenism and splenic sequestration - the spleen, enlarged due to portal hypertension, can sequester and destroy blood cells, including platelets. However, the count, while lower, is not dangerously low - bleeding usually occurs when the platelet count falls below 20,000/mm³. CONCLUDING: Applying our clinical reasoning to the case, we can identify that the liver's inability to produce clotting factors due to the patient's alcohol-induced cirrhosis is causing his prolonged bleeding time, manifested in his elevated PT and INR. RESPONSE: Consequently, the mechanism that most increases this patient's risk for bleeding would be (A) Decreased production of factor VII. This contributes to the lack of blood clotting ability, increasing the patient's risk of continued gastrointestinal bleeding.

**AI_E:** RESTATING THE QUESTION: The query is seeking to identify the principal mechanism that heightens the likelihood of bleeding in a patient characterized by lower gastrointestinal bleeding, a history of alcohol-induced cirrhosis, marked ascites, splenomegaly, and certain laboratory results, such as prolonged PT and elevated INR, as well as reduced platelet count. The patient's lab results and physical exam findings suggest a disrupted hemostasis (blood clotting process), leading to a higher risk for bleeding. Understanding these mechanisms requires knowledge in the domain spaces of pathophysiology, pathology, and hematology. PLANNING: The assumptions made here involve an understanding of the effects on the hemostatic system, specifically related to the liver's functions due to cirrhosis and its repercussions on clotting factors and other variables at play. For example, the liver's role in synthesizing clotting factors like factor VII (Option A) and in the metabolism of vitamin K necessary for several clotting factors (Option D), the function of platelets in clot formation and how their aggregation could be affected (Option B), the role of thrombopoietin in stimulating platelet production (Option C), and the possible effects of splenomegaly on platelet sequestration (Option E) need to be taken into account. The main mechanism should be one that would directly drive the clinical symptoms, i.e., lower gastrointestinal bleeding. CLINICAL REASONING: Alcohol-induced cirrhosis disrupts normal liver function and architecture. This condition impairs the synthesis of clotting factors including factor VII (Option A), which would lead to a prolongation of the PT and elevated INR as seen in the patient. Additionally, cirrhosis can impact vitamin K metabolism and subsequent synthesis of its dependent clotting factors (Option D). However, alcohol-related liver disease more commonly causes coagulopathy primarily by reducing the hepatic production of coagulation factor than by vitamin K dysfunction. In regard to platelets (Options B and C), while cirrhosis can induce functional alterations, these changes generally do not result in significant bleeding risk unless the platelet count is severely low (<50,000/mm³), which is not the case with our patient. Thrombopoietin, primarily made in the liver, is indeed decreased in cirrhosis, potentially reduced platelet production. Yet again, this mechanism would come into play in cases of profound thrombocytopenia. Splenomegaly and subsequent splenic sequestration (Option E), common in cirrhosis due to portal hypertension, leads to pooling and storage of up to 90% of the body's platelets, significantly reducing their availability for hemostasis. CONCLUDING: After examining each mechanism, the most likely, given the patient's clinical presentation and laboratory results, is the decreased production of clotting factors in the liver. The elevated PT and INR, indicators of abnormal extrinsic and common coagulation pathways usually seen in liver disease, suggest a reduction in production or functionality of clotting factors. The moderately decreased platelet count could be a contributing factor, but not severe enough to be the main reason. RESPONSE: The mechanism that most increases this patient's risk for bleeding is (A) Decreased production of factor VII.

**Facilitator** **[Round 2]:** Summary: AI_A believes that the primary mechanism contributing to the patient's increased risk of bleeding is the decreased production of factor VII due to the patient's alcohol-induced cirrhosis. This is evidenced by the patient's prolonged PT and INR, which suggest a problem with the liver's ability to produce clotting factors. AI_B, while acknowledging the potential role of decreased production of factor VII, argues that the primary mechanism is the inhibition of vitamin K epoxide reductase. This is due to chronic alcoholism leading to diminished absorption of vitamin K in the gut, impacting the synthesis of vitamin K-dependent clotting factors, including factor VII, in the liver. AI_C suggests that the primary mechanism is splenic sequestration, where the enlarged spleen sequesters too many platelets, lowering the overall number available to help stop bleeding. This is in addition to the liver's decreased capability of producing clotting factors. AI_D agrees with AI_A, stating that the primary mechanism is the decreased production of factor VII due to liver damage from alcohol-induced cirrhosis. This is supported by the patient's prolonged PT and INR, which suggest a defect in the clotting cascade. AI_E also agrees with AI_A and AI_D, stating that the primary mechanism is the decreased production of factor VII. This is due to the disruption of normal liver function and architecture from alcohol-induced cirrhosis, impairing the synthesis of clotting factors. Question for Council Members: Given the differing opinions on the primary mechanism contributing to the patient's increased risk of bleeding, could you please elaborate on the interplay between decreased production of factor VII, inhibition of vitamin K epoxide reductase, and splenic sequestration in the context of this patient's alcohol-induced cirrhosis? How do these mechanisms interact and contribute to the patient's condition, and what evidence can be used to determine which mechanism is most significant?. Based on your preceding response, how would you answer the original question: [A 47-year-old man is admitted to the intensive care unit because of lower gastrointestinal bleeding. He has a history of alcohol-induced cirrhosis. Physical examination shows marked ascites and splenomegaly; the spleen tip is palpated 4 cm below the left costal margin. Results of laboratory studies are shown: Serum Blood Creatinine 1.5 mg/dL Hematocrit 29% Hemoglobin 9.7 g/dL Platelet count 105,000/mm³ PT 25 seconds INR 2.5 Which of the following mechanisms most increases this patient’s risk for bleeding? (A) Decreased production of factor VII (B) Defective platelet aggregation (C) Deficiency of thrombopoietin (D) Inhibition of vitamin K epoxide reductase (E) Splenic sequestration]

**AI_B:** Responses: AI_A asserts that decreased production of factor VII is the most significant factor in the patient's bleeding due to his liver's diminished ability to synthesize clotting factors because of cirrhosis. AI_B, while acknowledging the potential role of decreased production of factor VII, believes that the inhibition of vitamin K epoxide reductase is more impactful. This enzyme's inhibition could lead to decreased synthesis of vitamin K-dependent clotting factors, including factor VII. This should be considered in a patient with alcohol-induced cirrhosis where chronic alcohol ingestion can lead to malabsorption and subsequent deficiency of fat-soluble vitamins like vitamin K. AI_C believes that splenic sequestration is the leading cause, where the enlarged spleen takes up and stores too many platelets, lowering the overall number available to help stop bleeding. However, it is important to note that this mechanism can co-exist with the reduced production of clotting factors due to cirrhosis affecting the function of the liver. AI_D agrees with AI_A, focusing on the decreased production of factor VII due to liver damage, causing a decreased production of clotting factors, which is evidenced by the prolonged prothrombin time (PT) and international normalized ratio (INR). AI_E also supports decreased production of factor VII as the primary mechanism behind the increased bleeding risk. It is due to disruption of normal liver function from alcohol-induced cirrhosis that impairs the synthesis of clotting factors. Conclusions: Upon collectively evaluating our responses, it seems that the council's major inclination is towards ubiquitous liver dysfunction, more specifically the decreased production of factor VII caused by cirrhosis, due to long-standing alcohol abuse. This is evidenced by the patient's prolonged PT and INR. It is crucial to note that all the mechanisms mentioned can co-exist and contribute to the patient's condition, but based on available information, decreased production of clotting factors seem to play a chief role. Final Response: So, to answer the original question, the mechanism most likely to be increasing this patient's risk for bleeding is (A) decreased production of factor VII due to the alcohol-induced cirrhosis affecting the liver's function in synthesizing clotting factors.

**AI_E:** Firstly, let's rephrase the question for understanding: The asked query is about the predominantly contributing factor to the increased risk of bleeding in a 47-year-old male patient with alcoholic cirrhosis. The physical condition of the patient reveals ascites and enlarged spleen. Laboratory outcomes provide insights about blood creatinine, hematocrit, Hemoglobin, platelet count, PT, and INR. Among the options provided, our task is to select the one that justifies best the patient's condition. Digging deeper into each assumption, we get: 1. Decreased production of factor VII (assumption A): With cirrhosis, there's impaired function or loss of hepatic cells that produce crucial factors for the clotting cascade, including factor VII. The presented higher International Normalized Ratio (INR) and prothrombin time (PT) suggest a malfunction in the clotting cascade, likely due to an issue with factor production. 2. Inhibition of vitamin K epoxide reductase (assumption D): Chronic and heavy alcohol consumption can cause malabsorption, including vitamin K, in-turn affecting the synthesis of vitamin K-dependent clotting factors in the liver. However, while it might contribute, it's usually not the predominant cause in a bleeding disorder in cirrhosis patients. 3. Splenic sequestration (assumption E): Enlarged spleen evident in splenomegaly can sequester platelets and lower the platelet count, ironically, contributing towards bleeding risks. However, the presented platelet count is above 50,000/mm³, at which point spontaneous bleeds occur. Surveying these critical assumptions, assumption A (decreased production of factor VII) appears to be the primary mechanism contributing to the patient's bleeding disorder given the evidence of dysfunction in the clotting cascade and this concept should be clinically prioritized. It should be noted that cirrhosis is a multisystem disorder, and thus multiple factors are likely contributing to the patient's bleeding risk including all assumptions stated above but among those, factor VII deficiency (due to impaired function or loss of hepatic cells) appears to be more prominent. Therefore, concluding, the predominant mechanism increasing this patient's risk of bleeding is (A) Decreased production of factor VII. It's vital to note that the other alternatives are contributing factors. They are not, however, mainly responsible for the high bleeding risk observed. This conclusion aligns with the clinical reasoning presented, connecting the analytical assessment of the alternatives available. Still, further investigations are needed for complete risk assessment.

**AI_C:** 1) RESTATING THE QUESTION: The question seeks to understand the major mechanisms that could be amplifying the risk of bleeding in a patient with known alcohol-induced cirrhosis, diagnosed lower gastrointestinal bleeding, and presenting remarkable ascites, splenomegaly, and lab results revealing a combination of anemia and coagulation abnormalities. 2) PLANNING: The initial evidence presented in this case indicates a problem with coagulation, which could be attributed to various possible mechanisms - specifically: (A) Decreased production of factor VII, (B) Defective platelet aggregation, (C) Deficiency of thrombopoietin, (D) Inhibition of vitamin K epoxide reductase, (E) Splenic sequestration. Upon evaluation of these assumptions, it seems plausible to explore decreased coagulation factor production due to cirrhosis and splenic sequestration as primary potential issues given the specific findings of the case. 3) CLINICAL REASONING: Alcohol-induced cirrhosis often leads to impaired liver function, including decreased production of coagulation factors. This decrease could explain the abnormal Prothrombin Time (PT) and International Normalized Ratio (INR) seen in this patient, which are indicators of the extrinsic and common coagulation pathways where factor VII plays a major role. On the other hand, splenic sequestration could also contribute to the patient's increased risk for bleeding, as an enlarged spleen can sequester or 'hold' too many platelets, reducing their availability in the bloodstream. This circumstance can contribute to thrombocytopenia, or a reduced platelet count, which could affect hemostasis. 4) CONCLUDING: Considering the presented symptoms, lab results, and known complications with alcohol-induced cirrhosis, decreased production of coagulation factors due to liver impairment, coupled with splenic sequestration leading to thrombocytopenia, seems to be the likely combination driving the increased bleeding risk in this patient. 5) RESPONSE: As per the aforementioned clinical reasoning process, out of the provided options, the mechanism that most increases this patient’s risk for bleeding is likely (A) Decreased production of factor VII. This is due to the impaired liver function from cirrhosis, reducing its ability to produce coagulation factors, including factor VII, that are necessary for normal blood clotting. Nevertheless, the simultaneous occurrence of splenic sequestration should not be overlooked since it weakens the overall hemostatic mechanism by reducing platelet count.

**AI_A:** Restatement: The council has been asked to consider a patient's story about his increased risk of bleeding and the primary cause of this complication in the context of alcohol-induced cirrhosis. Various contributors to this condition, including decreased production of factor VII, the impairment of vitamin K epoxide reductase, and splenic sequestration are given consideration. Given the patient's laboratory results, such as the noticeable rise in PT and INR, an increased risk for bleeding may likely be due to one or more anomalies in the coagulation pathway. Planning: To better address this scenario, I'm assuming that the alcohol-induced cirrhosis has impaired the man's liver function and has further affected his coagulation factors production, platelet count, and portal hypertension leading to splenomegaly and sequestration. Despite this, the question is whether the primary driving factor of his increased bleeding risk is due to a reduction in Factor VII (a vitamin K-dependent factor), an inhibition in vitamin K epoxide reductase, or from splenic sequestration due to an enlarged spleen. Clinical Reasoning: The man's report suggests he has portal hypertension (marked ascites and splenomegaly), a common complication of cirrhosis. Portal hypertension can cause splenomegaly and splenic sequestration, which would lower platelet count (thrombocytopenia). However, the man's platelet count isn't excessively low, which doesn't fully support splenic sequestration being the primary cause. The PT and INR are coagulation tests that evaluate the extrinsic and common pathways of the coagulation cascade, which comprise factors VII, X, V, II (prothrombin), and I (fibrin). Their prolongation, observed in the patient, would suggest potential issues with one or more of these factors. Both the decreased production of factor VII and inhibition of vitamin K epoxide reductase could lead to such prolongation since factor VII is a vitamin K-dependent factor made in the liver. Alcohol-induced cirrhosis can impair liver function and potentially decrease production of coagulation factors. However, chronic alcoholism can also disrupt the gut's ability to absorb vitamin K, leading to a deficit and thereby affecting the synthesis of all vitamin K-dependent coagulation factors (II, VII, IX, X) in the liver. This could lead to a greater risk of bleeding as compared to just the decrease in a single factor (factor VII). Concluding: Thus, taking into consideration of the patient's lab results and the greater impact of a deficit in vitamin K absorption due to chronic alcoholism, the inhibition of vitamin K epoxide reductase appears to be a more plausible contributor to the patient's increased risk of bleeding over a simple decrease in the production of factor VII or splenic sequestration. Response: Based on the above clinical reasoning and review of the proposed mechanisms, I would answer the question as (D) Inhibition of vitamin K epoxide reductase most increases this patient's risk for bleeding.

**AI_D:** Restating the question: The question is asking which of the given options contributes the most to the increased bleeding risk for a patient diagnosed with alcohol-induced cirrhosis, who presents with lower GI bleeding, ascites, splenomegaly, reduced platelet count, abnormal PT and INR values. Planning: There are different assumptions to be discussed here. Alcohol-induced cirrhosis reduces the liver's ability to synthesize and secrete vital substances, including clotting factors like Factor VII. However, it can also lead to decreased production and absorption of Vitamin K in the gut, which is essential for the synthesis of Vitamin K-dependent clotting factors. Also, an enlarged spleen can sequester platelets leading to thrombocytopenia and increase the patients' risk of bleeding. It will be essential to consider the effects of these mechanisms in light of the given lab results and physical findings to prioritize the most clinically significant contributor to the patient's status. Clinical reasoning: Patients with alcohol-induced cirrhosis typically show a decrease in liver function and an increase in bleeding risk due to deficiencies in clotting factors. A prolonged PT, as seen in this patient, often indicates a decrease in the liver's production of clotting factors, including Factor VII, which is one of the primary factors assessed by this test. The extended INR value also indicates an issue with extrinsic clotting pathway, which involves factor VII. The patient's decreased platelet count could be a direct consequence of splenic sequestration due to splenomegaly as suggested by the physical examination. Last, inhibition of Vitamin K epoxide reductase decreases the regeneration of Vitamin K, leading to reduced synthesis of Vitamin K-dependent clotting factors, including factor VII, in liver. Notwithstanding, this inhibition is unlikely in this case as it is commonly associated with warfarin use, which isn't indicated here. Concluding: In understanding the key contributors to the patient's bleeding risks, the decreased production of clotting factors particularly Factor VII due to alcohol-induced cirrhosis is a prominent possibility. Even though the inhibition of Vitamin K epoxide reductase and splenic sequestration can play a role, the PT and INR results manifest a more significant deficiency in clotting mechanisms. As the liver is the primary source of clotting factors, it is likely that the reduced liver function has a significant effect on the clotting mechanism, leading to an increase in the patient's risks of bleeding. Response: Therefore, based on the presented information and clinical reasoning, the decreased production of Factor VII (Option A) could be regarded as the primary mechanism increasing this patient's risk for bleeding in the context of alcohol-induced cirrhosis. Of course, other factors like inhibition of Vitamin K epoxide reductase and splenic sequestration may also be contributing, and their implications should be considered in the wider management of the patient's condition.

**Facilitator** **[Round 3]:** Summary: AI_A believes that the inhibition of vitamin K epoxide reductase is the primary contributor to the patient's increased risk of bleeding, given the patient's lab results and the greater impact of a deficit in vitamin K absorption due to chronic alcoholism. AI_B, AI_D, and AI_E all agree that the decreased production of factor VII due to alcohol-induced cirrhosis affecting the liver's function in synthesizing clotting factors is the primary mechanism behind the increased bleeding risk. They argue that the prolonged PT and INR, which are indicators of the extrinsic and common coagulation pathways where factor VII plays a major role, support this conclusion. AI_C suggests that both decreased production of coagulation factors due to liver impairment and splenic sequestration leading to thrombocytopenia are likely contributing to the increased bleeding risk in this patient. However, AI_C also leans towards decreased production of factor VII as the primary mechanism. Key Differences: The key differences in the council members' perspectives lie in the weight they assign to different potential causes of the patient's increased bleeding risk. AI_A places more emphasis on the inhibition of vitamin K epoxide reductase due to chronic alcoholism, while AI_B, AI_D, and AI_E focus more on the decreased production of factor VII due to liver damage from cirrhosis. AI_C considers both these factors but leans towards the decreased production of factor VII. Question for Clarification: Given the differing perspectives, a question that could help clarify the disagreements between the council members' opinions could be: "How do you weigh the impact of decreased production of factor VII versus the inhibition of vitamin K epoxide reductase in contributing to the patient's increased risk of bleeding, and how do the patient's lab results influence your assessment?". Based on your preceding response, how would you answer the original question: [A 47-year-old man is admitted to the intensive care unit because of lower gastrointestinal bleeding. He has a history of alcohol-induced cirrhosis. Physical examination shows marked ascites and splenomegaly; the spleen tip is palpated 4 cm below the left costal margin. Results of laboratory studies are shown: Serum Blood Creatinine 1.5 mg/dL Hematocrit 29% Hemoglobin 9.7 g/dL Platelet count 105,000/mm³ PT 25 seconds INR 2.5 Which of the following mechanisms most increases this patient’s risk for bleeding? (A) Decreased production of factor VII (B) Defective platelet aggregation (C) Deficiency of thrombopoietin (D) Inhibition of vitamin K epoxide reductase (E) Splenic sequestration]

**AI_C:** Restatement: We are trying to determine the primary cause of an increased risk of bleeding in a 47-year-old man with alcohol-induced cirrhosis. Crucial factors such as serum creatinine, blood hematocrit, hemoglobin, platelet count, PT, and INR were provided along with findings from a physical examination. Planning: The assumptions are that the result of his lab studies and physical examination are sufficient to make a diagnosis. We would want to challenge this by considering various potential causes, such as decreased production of factor VII, defective platelet aggregation, deficiency of thrombopoietin, inhibition of vitamin K epoxide reductase, or splenic sequestration. However, considering the history of alcohol-induced cirrhosis and the laboratory results with extended PT and raised INR, the clinically prioritized assumption would likely be decreased production of factor VII. Clinical Reasoning: The liver, being the primary site for the synthesis of most coagulation factors including factor VII, might be adversely affected due to alcohol-induced cirrhosis. Decreased production of factor VII, a vitamin K-dependent clotting factor, would disturb the extrinsic pathway leading to prolonged PT and raised INR, both of which have been noted in this individual's laboratory results. Concluding: After careful assessment, it can be stated that the alcohol-induced liver cirrhosis has likely affected the synthesis of clotting factors including factor VII, leading to an increased risk of bleeding. This conclusion is further supported by the lab results showing prolonged PT and raised INR. Given the above explanation, the best response to the original question would be: (A) Decreased production of factor VII.

**AI_D:** Restating the Question: The problem statement asks for the primary mechanism leading to a higher risk of bleeding in a 47-year-old male with a history of alcohol-induced cirrhosis, presenting with lower gastrointestinal bleeding. The patient also exhibits signs of splenomegaly and marked ascites. Laboratory results tell us that the patient has an elevated PT and INR, indicating a disorder in the coagulation system, and a lowered platelet count. Planning: While considering the risk for bleeding, assumptions can be based on his clinical features and lab results. Cirrhosis could lead to decreased production of clotting factors like Factor VII because the liver is its primary production site. Alcoholism could potentially result in defective absorption of vitamin K, leading to its reduced bioavailability for coagulation factors synthesis, thereby impairing the Vitamin K dependent epoxide reductase. Splenomegaly could cause an increased sequestration of platelets, resulting in thrombocytopenia. Each of these assumptions correlates with an increased risk for bleeding, but the clinical manifestation's primary driver needs to be identified. Clinical Reasoning: The patient presented with lower gastrointestinal bleeding, cirrhosis, and an elevated PT and INR. Given the history of alcohol-induced cirrhosis, there would be a decreased production of clotting factors, including Factor VII. This would result in a prolonged PT and INR. Despite the plausible assumption of vitamin K epoxide reductase inhibition, the PT/INR elevation leans the clinical picture more towards a decreased production of clotting factors including Factor VII. Concluding: Clinically, the patient's presentation and symptoms would most likely be increased due to a decreased production of Factor VII because of the role the liver plays in coagulation, and its function is compromised due to the patient's cirrhosis. The prolonged PT and INR also act as indicators of diminished Factor VII activity. Response: The mechanism most increasing this patient’s risk for bleeding is likely (A) Decreased production of factor VII.

**AI_E:** RESTATING THE QUESTION: The question is asking about the most significant mechanism that's increasing the risk of bleeding in a middle-aged male patient. His medical history includes alcohol-induced cirrhosis, ascites, and splenomegaly. His laboratory test results show signs of decreased hemoglobin and hematocrit, platelet count slightly below normal, and increased prothrombin time (PT) and international normalized ratio (INR), clinical findings suggestive of a coagulation disorder. PLANNING: The assumptions made here revolve around the patient's coagulopathy and its potential causes. Possibilities include decreased production of factor VII, defective platelet aggregation, deficiency of thrombopoietin, inhibition of vitamin K epoxide reductase, or splenic sequestration. Each contributes to the overall coagulopathy but to varying extents. CLINICAL REASONING: Cirrhosis often results in a reduction in the production of clotting factors by the liver, including factor VII. This affects the extrinsic and common pathways of coagulation, reflected by the prolonged PT and INR. Even though alcohol use inhibits vitamin K epoxide reductase, this wouldn't affect factor VII levels as factor VII isn't vitamin K dependent. Defective platelet aggregation usually has normal platelet counts, this isn't the case here. Thrombopoietin deficiency might cause thrombocytopenia, but it wouldn't cause a prolonged PT and INR. The splenomegaly hints towards splenic sequestration of platelets (partial thrombocytopenia), yet this doesn't fully explain the abnormal coagulation markers. CONCLUDING: Considering these findings, the clinical picture points more towards decreased production of factor VII due to cirrhosis. Firstly, this fits with the patient's documented chronic liver disease. Secondly, it explains both the thrombocytopenia (via splenic sequestration) and notably the prolongation of PT and INR, which involves factor VII in their pathways. RESPONSE: Thus, the principle mechanism increasing this patient's risk for bleeding is likely (A) Decreased production of factor VII.

**AI_A:** RESTATING THE QUESTION: This scenario discusses a 47-year-old man hospitalized in the intensive care unit due to lower gastrointestinal bleeding. He has a history of alcohol-induced cirrhosis. His physical examination reveals significant ascites and splenomegaly, with the spleen tip palpable 4 cm below the left costal margin. Lab studies present abnormal results, such as low hematocrit and hemoglobin, reduced platelet count, and prolonged PT and INR. The inquiry focuses on identifying the most contributory mechanism to the patient's increased risk for bleeding among the given choices. PLANNING: In evaluating the possible mechanisms for the increased bleeding risk in this patient, we can assess each factor, including the implications of decreased production of Factor VII, defective platelet aggregation, deficiency of thrombopoietin, inhibition of vitamin K epoxide reductase, and splenic sequestration. However, we should prioritize the factors relating to the patient's known alcohol-induced cirrhosis and its typical repercussions that contribute to bleeding risk. CLINICAL REASONING: In relation to his cirrhosis, the patient's liver function is likely impaired. The liver's role in the synthesis of coagulation factors, specifically Factor VII, and its processing of vitamin K is crucial to consider. With liver function compromised due to cirrhosis, decreased production of Factor VII (A) could indeed be impacting the patient's coagulation, indicated by the prolonged PT and INR noted in the lab results. Simultaneously, chronic alcoholism is well-known for leading to malnutrition, particularly vitamin K deficiency, which can cause inhibition of vitamin K epoxide reductase (D). However, compared to the detrimental effects of cirrhosis on Factor VII synthesis, the impact of alcohol on vitamin K absorption might be less significant in this case. CONCLUDING: After iterating through the clinical reasoning process, we infer that the decreased production of Factor VII due to alcohol-induced cirrhosis of the liver, with its subsequent impact on the synthesis of clotting factors, could predominantly contribute to the increased risk of bleeding in this patient. RESPONSE: Therefore, in this case, the most prevailing mechanism that increases this patient's risk for bleeding corresponds to decreased production of factor VII (A).

**AI_B:** Restatement of the question: We are being asked to identify the main factor that is contributing to the increased risk of bleeding in a 47-year-old male patient with alcohol-induced cirrhosis, who shows lab results including decreased hematocrit and hemoglobin levels, a lower platelet count, and elevated Prothrombin Time (PT) and International Normalized Ratio (INR). These lab results suggest issues related to clotting and blood volume regulation. Planning: Bleeding in this patient could be due to several factors such as decreased production of factor VII, Defective platelet aggregation, deficiency of thrombopoietin, inhibition of vitamin K epoxide reductase, or splenic sequestration. I would prioritize the evaluation of this patient's coagulation factor levels, as these could be directly influenced by the patient's liver condition, which is known to be impaired in individuals with cirrhosis. Clinical reasoning: Given the patient's history of alcohol-induced cirrhosis, it is plausible that the liver's ability to synthesize crucial clotting factors, such as factor VII, is compromised. This could be validated with the prolonged PT and INR seen in this patient, as these are measures of the extrinsic and common coagulation pathways where factor VII is instrumental. Additionally, chronic alcoholism can inhibit the absorption of vitamin K, which can result in a defect in the synthesis of vitamin K dependent clotting factors, including factor VII. This would further exacerbate the bleeding tendency. Concluding: Conclusively, while inhibition of vitamin K epoxide reductase, defective platelet aggregation, deficiency of thrombopoietin, and splenic sequestration may all play crucial roles in the development of bleeding in this patient, given the patient's medical history and lab findings, it would be most logical to assume that decreased production of factor VII due to liver impairment is the primary contributor to his increased bleeding risk. Response: Hence, based on the clinical thought process outlined above, my response to the question would be (A) Decreased production of factor VII. This assumption is mainly based on the patient's history of alcohol-induced cirrhosis and the lab findings, particularly the prolonged PT and INR, which suggest the possibility of a decrease in factor VII, a key component of the extrinsic and common coagulation pathways.

**Facilitator** The consensus statement synthesizes the responses of AI_A, AI_B, AI_C, AI_D, and AI_E as follows: The scenario presents a 47-year-old man with alcohol-induced cirrhosis, admitted to the intensive care unit due to lower gastrointestinal bleeding. His physical examination reveals significant ascites and splenomegaly, and his lab studies show abnormal results such as low hematocrit and hemoglobin, reduced platelet count, and prolonged PT and INR. The task is to identify the primary mechanism contributing to the patient's increased risk for bleeding. In evaluating the possible mechanisms, we consider factors such as decreased production of Factor VII, defective platelet aggregation, deficiency of thrombopoietin, inhibition of vitamin K epoxide reductase, and splenic sequestration. However, given the patient's history of alcohol-induced cirrhosis and the lab results, the focus is on the implications of the liver's impaired function on the synthesis of coagulation factors, specifically Factor VII, and its processing of vitamin K. The liver's role in the synthesis of coagulation factors is crucial, and with its function compromised due to cirrhosis, decreased production of Factor VII could be impacting the patient's coagulation, as indicated by the prolonged PT and INR noted in the lab results. Chronic alcoholism can also lead to malnutrition, particularly vitamin K deficiency, which can cause inhibition of vitamin K epoxide reductase. However, the impact of alcohol on vitamin K absorption might be less significant in this case compared to the detrimental effects of cirrhosis on Factor VII synthesis. Therefore, after a thorough clinical reasoning process, the consensus is that the decreased production of Factor VII due to alcohol-induced cirrhosis of the liver, with its subsequent impact on the synthesis of clotting factors, is the primary contributor to the increased risk of bleeding in this patient. This conclusion is further supported by the lab results showing prolonged PT and raised INR. Hence, the most prevailing mechanism that increases this patient's risk for bleeding corresponds to decreased production of factor VII.
